# Insights into *Streptomyces coelicolor* A3(2) growth and pigment formation with high‐throughput online monitoring

**DOI:** 10.1002/elsc.202100151

**Published:** 2022-04-28

**Authors:** Maurice Finger, Fabio Sentek, Lukas Hartmann, Ana M. Palacio‐Barrera, Ivan Schlembach, Miriam A. Rosenbaum, Jochen Büchs

**Affiliations:** ^1^ AVT ‐ Biochemical Engineering RWTH Aachen University Aachen Germany; ^2^ Leibniz Institute for Natural Product Research and Infection Biology Hans‐Knöll‐Institute Jena Germany; ^3^ Faculty of Biological Sciences Friedrich‐Schiller‐University Jena Germany

**Keywords:** high‐throughput, microtiter plate, online monitoring, pigmentation, *Streptomyces coelicolor*

## Abstract

*Streptomyces* species are intensively studied for their ability to produce a variety of natural products. However, conditions influencing and leading to product formation are often not completely recognized. Therefore, in this study, high‐throughput online monitoring is presented as a powerful tool to gain in‐depth understanding of the cultivation of the model organism *Streptomyces coelicolor* A3(2). Through online measurements of oxygen transfer rate and autofluorescence, valuable information about availability of nutrients and product formation patterns of the pigments actinorhodin and undecylprodigiosin can be obtained and explained. Therefore, it is possible to determine the onset of pigmentation and to study in detail the influencing factors thereof. One factor identified in this study is the filling volume of the cultivation vessel. Slight variations led to varying pigmentation levels. By combining optical and metabolic online monitoring techniques, the correlation of the filling volume with pigmentation could be explained as a result of different growth trajectories caused by varying specific power inputs and their influence on the pellet formation of the filamentous system. Finally, experiments with the addition of supernatant from unpigmented and pigmented cultures could highlight the applicability of the presented approach to study quorum sensing and cell‐cell interaction.

**Abbreviations**
LNP: low nitrogen phosphate
MTP, microtiter plate; OTR: oxygen transfer rate

## 1 INTRODUCTION

Soil‐dwelling, filamentous, and gram‐positive bacteria of the genus *Streptomyces* are well known for their capability to produce a wide variety of different bioactive secondary metabolites. Their application ranges from antibiotics over antivirals to antitumor agents and immunosuppressants [1, 2]. Biosynthesis of these compounds is often tied to complex regulatory mechanisms [3]. Identifying and triggering such mechanisms with defined laboratory conditions is a challenging task. In support, genetic investigations allow more profound insight into the metabolism of the cells and can assist in finding suitable production windows. However, although the complete genome of the model actinomycete *Streptomyces coelicolor* A3(2) was sequenced as early as 2002, several mechanisms of regulation are yet to be fully elucidated [4]. *S. coelicolor* serves as a model organism due to the formation of colored antibiotics. These are the tripyrollic and red‐pigmented prodigines such as undecylprodigiosin and the polyketidic, blue‐pigmented actinorhodin [5]. Nevertheless, even for these well studied compounds, a complete picture of all physiological control mechanisms is missing [6, 7]. The production is often activated once growth‐limiting conditions are reached and hence tied to an early stationary phase [8]. Growth limitations can either occur due to nutrient limitations such as the carbon; phosphate or nitrogen source; or environmental changes like temperature, pH, or osmolality. However, production and yields of the different secondary metabolites are affected differently by these limitations [9]. Furthermore, limitations are not always homogenous for all cells in a cultivation. Especially the morphology of filamentous organisms such as *S. coelicolor* affects limitations and therefore, productivity. The formation of pellets due to aggregation of hyphae and spores leads to diffusion gradients of nutrients along the pellet radius. Formation and density of pellets as well as the pellet radius are dependent on the occurring shear forces [10]. Hence, variations in power input can result in varying growth kinetics and productivity [11].

1 **PRACTICAL APPLICATION**

High‐throughput online monitoring through oxygen transfer rate and autofluorescence measurement is presented in this study as a powerful tool to understand intricate pigment producing microbes such as *Streptomyces coelicolor*. Pigmentation onset and intensity can be detected in the autofluorescence signal due to light absorption effects. These effects should especially be considered when other optical measurements are conducted. For example, pH or oxygen optode and fluorescence readouts for fluorescently tagged organisms can be heavily disturbed by spectral overlap of involved fluorophores and pigments. In contrast, at the example of pigmentation for *Streptomyces*, the advantage for natural product formation investigations is demonstrated. The application of high‐throughput online monitoring bears the potential for further studies regarding quorum sensing and cell‐cell interactions.

Antibiotic production is not only triggered by stress responses or growth cessation, but can be stimulated through small diffusible signaling molecules such as γ‐butyrolactones [12]. This signaling process is also called quorum sensing and enables cell‐cell communication. It is essential for a coordinated initiation of the production in the bacterial population. Hence, these molecules are referenced to as bacterial hormones [13]. *S. coelicolor* produces various γ‐butyrolactones, which have been reported to hold both activating and repressing effects on gene expression for antibiotic production [14, 15]. However, the experiments validating these findings are often performed on the genetic level or with cultivations on agar plates.

This study demonstrates that investigations of the pigmentation and therefore, antibiotic production behavior can be conducted in a high‐throughput online monitoring system with low effort. The technology paves the way for the examination and validation of influencing factors, which has the potential to significantly increase the speed of process development.

## 2 MATERIALS AND METHODS

### 2.1 Microorganisms

*Streptomyces coelicolor* A3(2) DSMZ40783 was received from the Hans‐Knöll‐Institute culture collection (Jena, Germany). All cultivations were inoculated with 10^6^ n_Spores_·mL^–1^. Spores were prepared similar to Hobbs et al. [16]. A spore suspension was spread on soy‐flour mannitol agar plates, which consisted of 20 g·L^–1^ mannitol, 20 g·L^–1^ soy‐flour and 20 g·L^–1^ agar. After 10‐day incubation at 30°C, the spores were harvested by scraping and suspending in deionized water. Mycelium was removed by filtration with a 40 µm cut‐off cell strainer (Corning, Corning, USA). Spore concentrations were adjusted in the stock solution to 10^8^ n_Spores_·mL^–1^ using a Multisizer 4 (Beckman Coulter, Brea, USA) coulter counter and stored at 4°C. The stock solution was vortexed for 30 s before inoculation of the medium to achieve an even distribution.

### 2.2 Media composition

Cultivations were performed in a low nitrogen phosphate (LNP) minimal medium similar to the medium previously adapted by Antonov et al. [17]. If not stated otherwise, this medium consisted of 30 g·L^–1^ glucose, 0.1 M 2‐(N‐morpholino)ethanesulfonic acid (MES), 3.0 g·L^–1^ (NH_4_)_2_SO_4_, 0.5 g·L^–1^ MgSO_4_·7 H_2_O, 0.4 g·L^–1^ KH_2_PO_4_, 0.3 g·L^–1^ urea, 0.23 g·L^–1^ CaCl_2_·2 H_2_O, 0.05 g·L^–1^ NaCl, 0.25 (v/v) % trace element solution, and 0.01 (v/v) % tween 80. The pH of the medium without trace elements was adjusted to 6.7 with 5 M NaOH. The trace element solution contains 180 g·L^–1^ citric acid, 16 g·L^–1^ ZnSO_4_·7 H_2_O, 2.71 g·L^‐1^ CoCl_2_·6 H_2_O, 2.29 g·L^–1^ Fe_2_(SO_4_)_3_, 2.05 g·L^–1^ CuSO_4_, 1.60 g·L^–1^ MnSO_4_·7 H_2_O, 0.8 g·L^–1^ H_3_BO_3_. All solutions were sterile‐filtered with a 0.2 µm cut‐off filter (MilliporeSigma, Burlington, USA).

### 2.3 Microtiter plate cultivations and online monitoring

For cultivations in microtiter plates (MTP), 48‐round well plates (MTP‐R48‐B, m2p‐labs GmbH, Baesweiler, Germany) were used. Unless stated otherwise, the filling volume was 1000 µL at a shaking frequency of 800 rpm with a shaking diameter of 3 mm and the temperature set to 30°C. Well resolved online monitoring of the oxygen transfer rate (OTR) was achieved with an in‐house built micro respiration activity online monitoring system [18]. In intervals of 16 min, microfluidic valves for aeration of the MTP wells were closed for 4 min. During this measurement time, the course of oxygen partial pressure was monitored in the headspace of the sealed wells. The change in oxygen partial pressure over time is approximated by a linear fit, with which the OTR can be calculated.

Autofluorescence wavelength (λ_ex_/λ_em_ 483/520 nm) measurements were performed in an in‐house built BioLector device coupled to a Fluoromax‐4 spectrometer (HORIBA Jobin‐Yvon GmbH, Bernsheim, Germany) [19]. The slit size for fluorescence measurements was 8 nm. For the integration time, 900 ms were chosen, which accounts for 12 rotations at 800 rpm. The intensity data of every experiment series was baseline corrected through normalization I_norm_ between 0 and 1 according to Equation (1). Additionally, this assisted a better comparability between experiments.

(1) $$I_{\mathrm{norm}}=\frac{I\;-\;\min_{\;}\left(I\right)}{\max_{\;}\left(I\right)\;-\underset{\;}{\;\min}\left(I\right)}\;$$

### 2.4 Shake flask cultivations and online monitoring

For cultivations in shake flasks, unbaffled 250 mL flasks were used. The filling volume was 20 mL at a shaking frequency of 350 rpm with a shaking diameter of 50 mm and the temperature set to 30°C. An in‐house built respiration activity monitoring system was used for online measurements of respiration activities in up to eight parallel shake flask [20, 21]. For each sample point, one flask was taken from of the system.

### 2.5 Offline measurements

Scattered light intensity scans (λ_ex_/λ_em_ 300–700 nm) after cultivation were performed with an excitation step size of 10 nm by an automated monochromator MicroHR Motorized (HORIBA Jobin Yvon GmbH, Unterhaching, Germany) and an emission resolution of the Synapse CCD detector (HORIBA Jobin Yvon GmbH, Unterhaching, Germany) of 0.44 nm. A detailed description of the setup was published by Ladner et al. [22]. Conditions during measurement were the same as cultivation conditions. The pH of samples, which were investigated for actinorhodin production, was increased to pH values larger than 12 by the addition of 10 (v/v) % 5 NaOH. The pH was measured using a pH‐meter HI 2211 (HANNA Instruments, Smithfield, USA).

Macroscopic pictures of culture broth samples were scanned using an Epson perfection V700 photo scanner (Epson, Tokyo, Japan). The image analysis of pictures was performed with ImageJ 1.53k [23]. For the determination of the projected pellet area and the maximum feret diameter, the 8‐bit version of the taken pictures was transformed into black and white pictures using the function “Auto threshold” on the region of interest. The function “Analyze particle” was applied for the same region to measure the projected pellet area and the maximum feret diameter. The mode red value of the cultivation broth and pellets was read out with the function “Histogram.” Therefore, either pellet free regions or pellets were chosen as region of interest until the mode could be determined from at least 5000 pixels.

## 3 RESULTS AND DISCUSSION

### 3.1 Correlations of online monitoring signals

**FIGURE 1:**
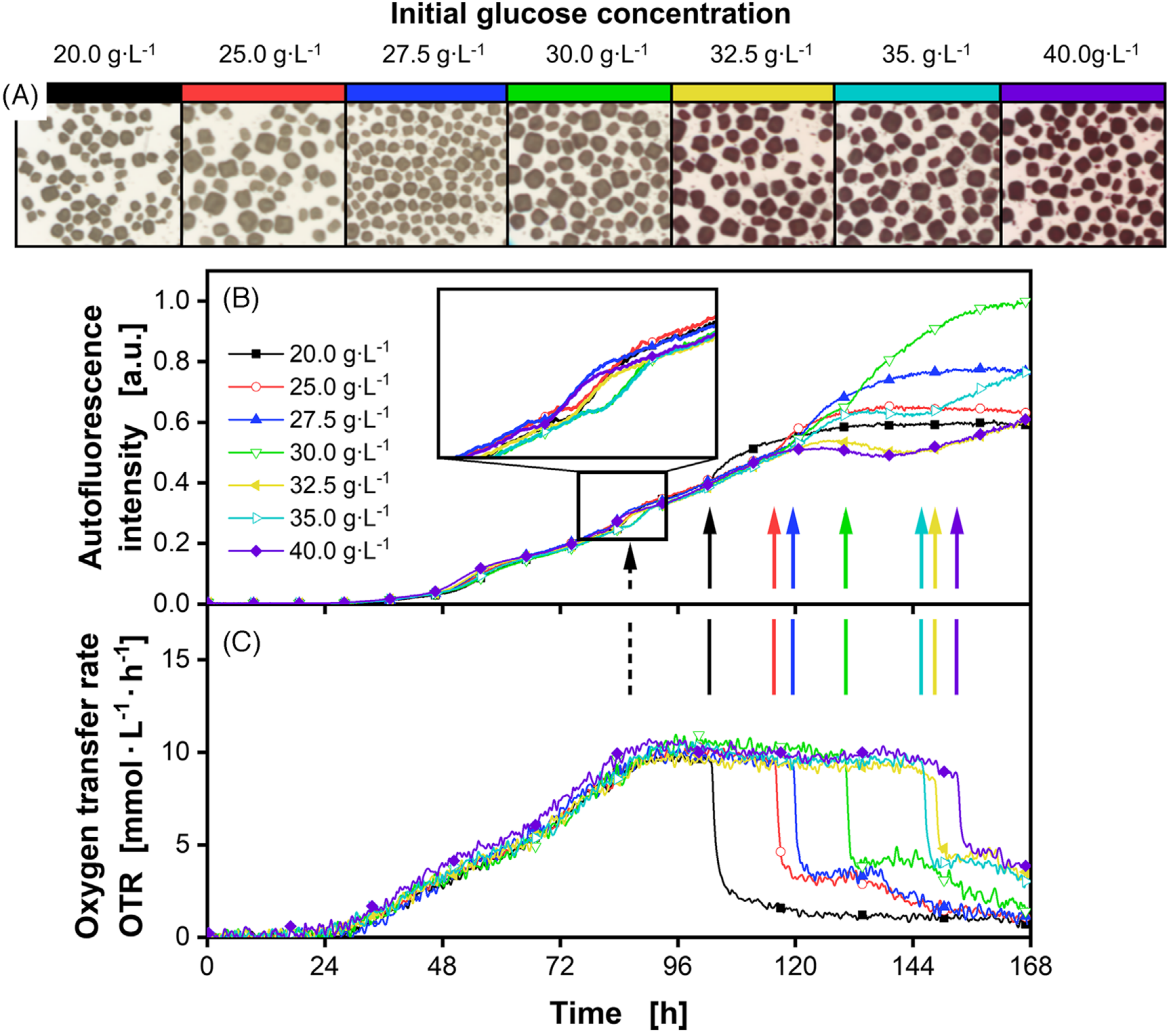
Cultivation of *Streptomyces coelicolor* A3(2) with varying glucose concentrations. (A) Macroscopic pictures of pellets after termination of the cultivation. The pellet size was in the order of 300 µm. (B) Normalized autofluorescence intensity signals (Excitation: 483 nm; Emission: 520 nm) with an enlarged view of the stress signal due to phosphate limitation. Marked by a dashed arrow. (C) Oxygen transfer rates. Solid arrows mark the glucose exhaustion. For clarity, only every 50th data point over time is indicated by the corresponding symbol in (B) and (C). Pictures shown in (A) and data presented in (B) and (C) originate from the same well for each condition, respectively. Culture conditions: 48‐well round well plate, V_L_ = 1000 µL, n = 800 rpm, d_0_ = 3 mm, T = 30°C, X_0_ = 10^6^ spores·ml^–1^, LNP medium with varying glucose concentrations

Initial experiments with *S. coelicolor* were conducted in shake flasks. The cultivations with 30 g·L^–1^ of glucose in a defined low nitrogen phosphate (LNP) medium were red‐pigmented after 168 h. Daily sampling from individual shake flasks indicated a rapid change in production of this red pigment (data not shown). Therefore, a detailed investigation implementing online monitoring using an optical system in MTPs was performed, to observe whether and how pigment formation can be monitored (Figure 1). The respiratory activity was tracked by measuring the OTR of each well. As emphasized in the literature, the OTR is linked to growth and can be used to determine growth characteristics as well as limitations [20, 24]. Especially, the depletion of the primary carbon source is well distinguishable in most cultivations by a rapid decrease in OTR. Autofluorescence of flavins was monitored at λ_ex_/λ_em_ 483/520 nm as an indicator for biomass and as a more precise signal compared to the scattered light signal [25, 26]. The autofluorescence depends on the organism and the formed metabolites, which can also be traced back to the metabolic state [26]. The duration of glucose availability and the resulting effects on pigmentation were studied by varying glucose concentrations from 20 to 40 g·L^–1^. Therefore, different endpoints for the cultivation from 104 to 154 h could be realized. The macroscopic pictures (Figure 1A) displayed a transition toward pigmentation at higher glucose concentrations. First signs of pigmentation were observed for a concentration of 30 g·L^‐1^ and higher. The autofluorescence indicates growth starting at 36 h (Figure 1B). However, according to the OTR, initial growth already begins at 24 h (Figure 1C), which can be explained by the higher sensitivity of this measurement technique. The OTR plateau starting at about 86 h appears due to a phosphate limitation, resulting from the low phosphate content in the LNP medium. This could be proven by sampling and measuring the phosphate concentration as well as by spiking a phosphate solution, which elevated the OTR plateau (Figure S1). The phosphate limitation can also be seen as a stress signal within the autofluorescence signal at 86 h (Figure 1B). The energy metabolism and therefore, the ratio of oxidized (fluorescent) to reduced (non‐fluorescent) flavins is drastically changing. Similar observations were made by Surre et al. [27]. They showed that stressing agents led to changes in NAD and FAD fluorescence [27]. Once respiratory activity drastically decreased due to the depletion of glucose, an increase of autofluorescence at precisely the same point in time is observed (Figure 1B–C solid arrows). The reason for this increase was not investigated in this study. However, for cultivations without phosphate limitation due to phosphate spiking (Figure S1) or the use of even lower amounts of glucose (Figure S2A), no increase after carbon source depletion can be noted. For the different glucose concentrations tested, the progression until depletion of glucose is similar, which showed a good reproducibility and a low effect of osmolality differences, resulting from different glucose concentrations. At higher glucose concentrations, the OTR plateau is prolonged and therefore, the increase of autofluorescence appeared later. However, all pigmented samples exhibited a different pattern, which is only visible in the autofluorescence and not in the OTR. At around 130 h, the intensities of all pigmented cultivations started dropping until the glucose was depleted and the autofluorescence rose again. Explanations for the decrease in autofluorescence at 130 h could be either a change in the energy metabolism, as explained before, or a reduction in excitation or emission intensity resulting from light absorption by the produced red pigment. This will be further evaluated in Section 3.3.

### 3.2 The influence of volumetric power input on growth kinetics and pigment production

**FIGURE 2:**
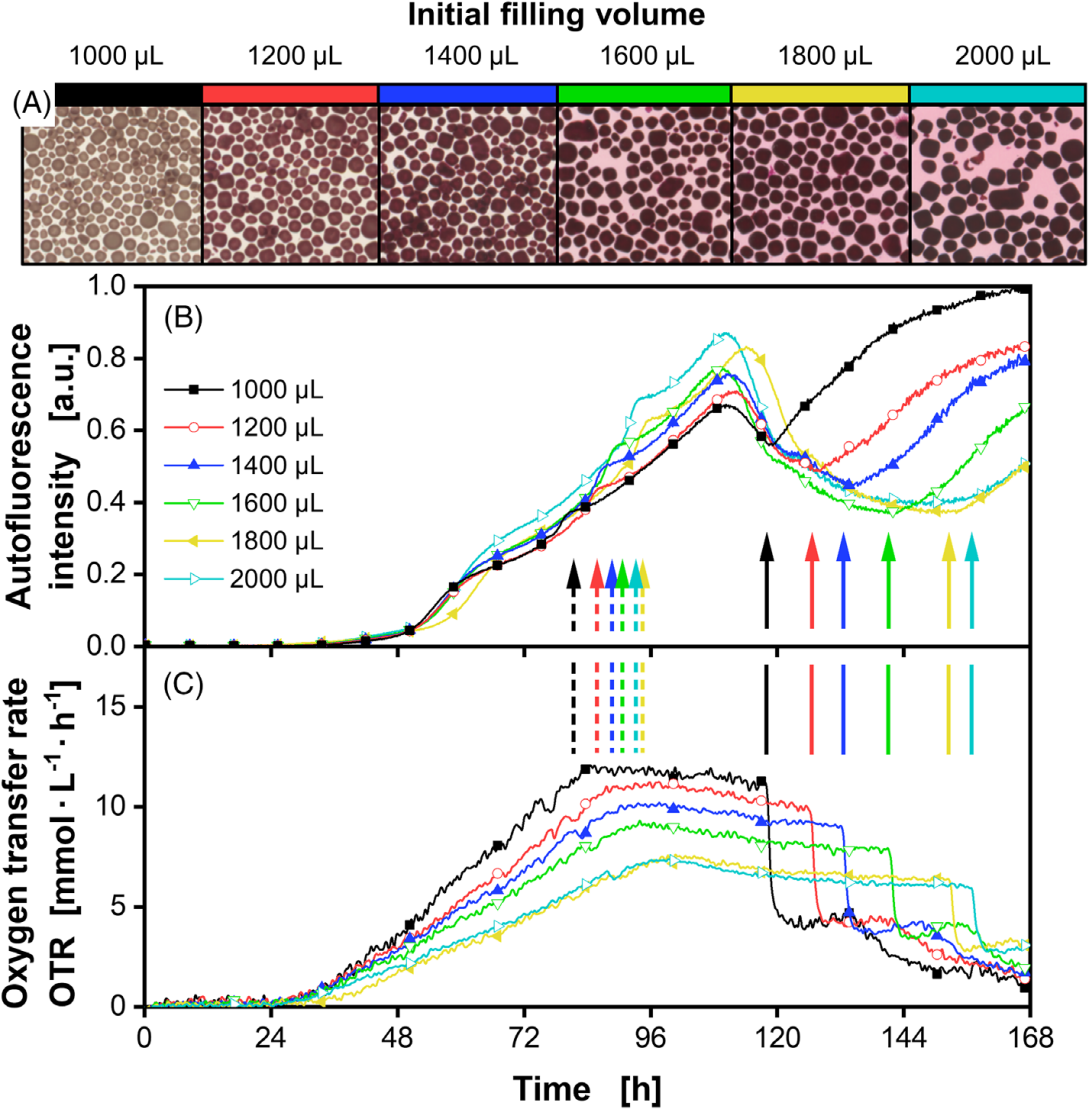
Cultivation of *Streptomyces coelicolor* A3(2) with varying filling volumes. (A) Macroscopic pictures of pellets after termination of the cultivation. The pellet size was in the order of 300 µm. (B) Normalized autofluorescence intensity signals (Excitation: 483 nm; Emission: 520 nm). (C) Oxygen transfer rates. Dashed arrows mark the stress signal due to phosphate limitation and solid arrows mark glucose exhaustion. For clarity, only every 50th data point over time is indicated by the corresponding symbol in (B) and (C). Pictures shown in (A) and data presented in (B) and (C) originate from the same well for each condition, respectively. Culture conditions: 48‐well round well plate, V_L_ = 1000–2000 µL, n = 800 rpm, d_0_ = 3 mm, T = 30°C, X_0_ = 10^6^ spores·mL^‐1^, LNP medium with 30 g·L^–1^ glucose

Cultivations of *Streptomyces* are subject to inherent complexity. Due to their filamentous nature and morphology, slight differences in cultivation conditions lead to significant changes in fermentation performance and especially in product formation [28]. Therefore, literature data is often hardly comparable and results differ in order of magnitudes. A parameter frequently not considered in small‐scale cultivations is the volumetric power input. This directly influences morphology, growth trajectories and therefore, alters product formation such as pigmentation [29]. The volumetric power input in MTP systems can mainly be influenced through shaking diameter, shaking frequency, or filling volume. Sohoni et al. as well as Koepff et al. studied how small‐scale cultivations of *Streptomyces* could be made more robust and reliable [30, 31]. Besides the investigation of the inoculum, the addition of glass beads and increase of shaking frequency were studied, respectively. Glass beads as well as a higher shaking frequency led to more homogenous and reproducible conditions. In this study, it is additionally shown that the choice of filling volume has a drastic effect on growth and pigment production (Figure 2). It is observed that with increasing filling volume, the slope of the OTR after 24 h of cultivation is decreasing. This can be explained by the concomitant reduction in volumetric power input and its impact on pelleting behavior (Figure 2A). The power input in 48‐well plates has not yet been characterized. However, Montes‐Serano et al. showed that for increasing filling volumes in 6‐, 24‐, and 96‐well plates a decrease in volumetric power input due to the change of surface to volume ratio can be noted [32]. The same effect holds true for 48‐well plates. The volumetric power input in 48‐well plates was roughly estimated with three different models (Figure S3) for shake flasks as well as for cylindrical shaken bioreactors [33, 34, 35]. With the increase in filling volume from 1000 to 2000 µL, a decrease of the volumetric power input of about 25%–37% can be calculated with all models. The decrease in volumetric power input led to fewer and bigger pellets. The pellet size was evaluated by determination of projected area (Figure S4A) and maximum feret diameter (Figure S4B) of pellets within the macroscopic images taken after the cultivation ended (Figure 2A). Both, the projected pellet area as well as the maximum feret diameter increase with increasing filling volumes. The median projected pellet area at the highest filling volume of 2000 µL is more than doubled with 0.19 mm^2^ compared to the median at the lowest filling volume condition of 1000 µL with 0.08 mm^2^. We hypothesize that in the early growth phase, a number of seed pellets are formed by spore aggregation depending on the power input, which later only grow in size but not in number. Similar results of an inversely related pellet number to pellet size was also previously reported by Tough and Prosser for varying impeller speeds and therefore, varying power input [10]. The linear OTR increase thereby shows the growth in pellet size, while the slope of the OTR increase is dependent on the seed pellet concentration. Interestingly, the effect is more pronounced with the addition of microparticles such as talc or cellulose (Figure S5A). The resulting change in growth culminated in different OTR plateau heights, as soon as the phosphate is depleted (Figure 2 dashed arrows). Hence, the OTR drop due to glucose depletion (Figure 2 solid arrows) for the lowest tested filling volume of 1000 µL occurred around 40 h earlier than for the highest of 2000 µL. The general autofluorescence trajectory is comparable to Figure 1. When phosphate becomes limiting, a stress signal is visible in the autofluorescence and the autofluorescence increases after glucose depletion (Figure S2B) in comparison to cultivations without phosphate limitation (Figure S2A). Furthermore, for pigmented cultivations a drop of the autofluorescence is visible (Figure S2C). With varying filling volume, the phosphate is depleted at different time points according to the OTR. Therefore, the stress signal in the autofluorescence is also shifted in time (Figure 2B and Figure S6 dashed arrows). The same holds true for the time point of glucose depletion. Interestingly, regardless of these nutritional effects, the start of pigment formation was highly synchronized under all conditions at around 110 h and was coincident with a clear decrease of the autofluorescence signal. Additionally, it is observed that the autofluorescence drops further down for higher filling volumes, where pigment formation was increased. This correlation is indicated by image analysis of the macroscopic images taken at the end of cultivation (Figure S7). The mode red value, which represents the red pigmentation level, of the cultivation broth (Figure S7A) as well as of the pellets (Figure S7B) correlates well with the autofluorescence value at the local minimum after the drop with R^2^ = 0.91 and R^2^ = 0.97. A correlation with the autofluorescence at the end of cultivation (t = 168 h) is also given (Figure S7). However, as the autofluorescence was still rising for the higher filling volume conditions, the coefficient of determination was lower with R^2^ = 0.80 and R^2^ = 0.89. The increased pigmentation with an increase in filling volume can be explained by the slower glucose consumption of these cultivations and therefore, extended time of glucose availability, which prolonged the pigment production phase. In conclusion, it can be stated that a higher filling volume directly leads to higher pigmentation levels (Figure 2A). The reason and factors affecting the synchronized pigmentation onset have yet to be determined, but will be further discussed in Section 3.4.

### 3.3 Light absorption effects caused by pigment formation

**FIGURE 3:**
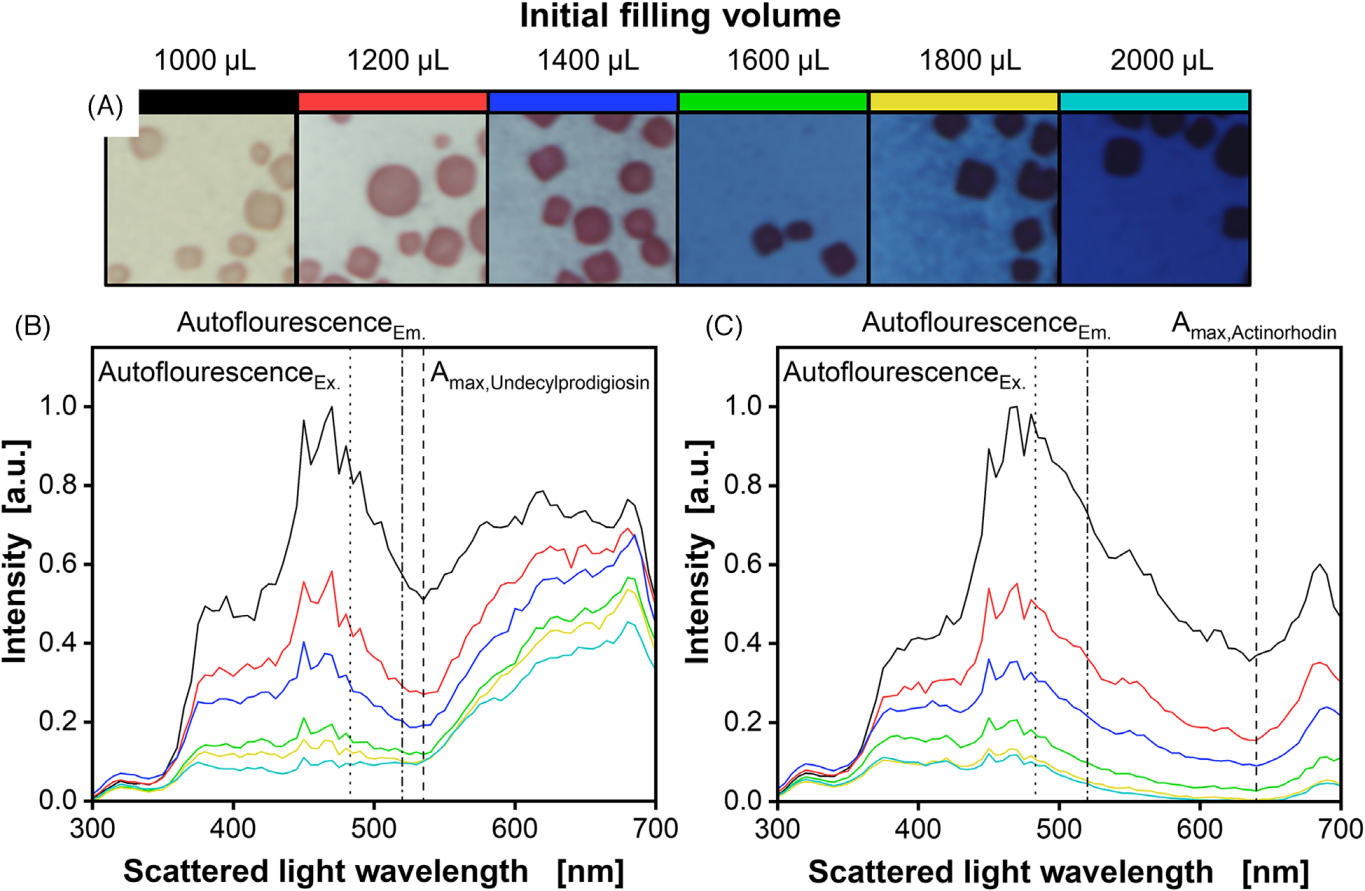
Pigmentation and resulting scattered light absorption of *Streptomyces coelicolor* A3(2) cultures with varied filling volumes. (A) Macroscopic pictures of pellets after pH were increased above pH 12. The pellet size was in the order of 300 µm. (B) Normalized scattered light intensity signals without pH adjustments. (C) Normalized scattered light intensity signals after pH was increased above pH 12. Dotted lines represent the excitation wavelength and dash‐dotted lines the emission wavelength for all autofluorescence measurements. Dashed lines indicate the maximum absorption wavelength of undecylprodigiosin and actinorhodin, respectively [37, 38]. Culture conditions: 48‐well round well plate, V_L_ = 1000–2000 µL, n = 800 rpm, d_0_ = 3 mm, T = 30°C, X_0_ = 10^6^ spores·ml^‐1^, LNP medium with 30 g·L^–1^ glucose

One requirement of optical online measurements is the fact that there should be little to no interference of the monitored variable or compound with others. Kunze et al. showed that this is not the case for many observed systems. For instance, an interference of mCherry fluorescent protein on the scattered light signal could be noted [36]. Therefore, to test whether the impact of pigmentation on autofluorescence is the result of a metabolic change or light absorption effects, pigmentation was investigated by spectrally resolved scattered light measurements at the end of the cultivation displayed in Figure 2. The scattered light spectra from 300 to 700 nm show a dip in the signal forming near the maximum absorption wavelength for undecylprodigiosin at 535 nm in Figure 3B [37]. This decreased intensity around the absorption maximum is systematically increasing with stronger pigmentation. The difference becomes even more evident in comparison with entirely unpigmented biomass (Figure S8). Since the autofluorescence is measured at an excitation wavelength of 483 nm (Figure 3B dotted line) and an emission wavelength of 520 nm (Figure 3B dash‐dotted line), the influence of pigmentation on autofluorescence can be deduced from the decreased scattered light intensity at this wavelength. Therefore, the decrease in autofluorescence noted in Figures 1B and 2B is clearly correlated to the light absorbing effects resulting from increasing pigmentation levels.

Besides undecylprodigiosin, *S. coelicolor* produces actinorhodin as well. However, this compound only turns to its blue color at alkaline conditions [38]. Hence, the pH of the samples after cultivation was increased to a pH > 12 by the addition of 10 (v/v) % 5 M NaOH solution. In the macroscopic pictures (Figure 3A), an increase of blue pigmentation with an increase in filling volumes and consequently a similar production pattern as for undecylprodigiosin can be perceived. Measurements of the scattered light spectra of these samples revealed a local minimum due to absorption at around 640 nm, which corresponds to the reported maximum absorption wavelength of actinorhodin (Figure 3C) [38].

### 3.4 Investigation of pigmentation onset

**FIGURE 4:**
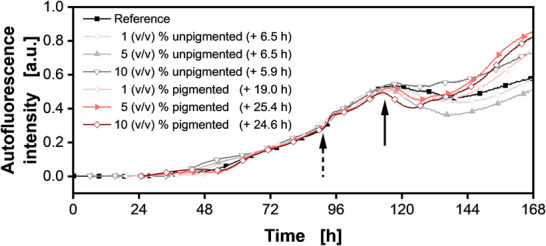
Cultivation of *Streptomyces coelicolor* A3(2) supplemented with different amounts of supernatants from unpigmented (gray scale) and pigmented (red scale) cultures. Normalized autofluorescence intensity signals (Excitation: 483 nm; Emission: 520 nm). Data was shifted according to the occurrence of the stress signal to the reference. For the unedited graph compare Figure S10. The dashed arrow marks the stress signal due to phosphate limitation and the solid arrow marks the pigmentation onset. For clarity, only every 50th data point over time is indicated by the corresponding symbol. Data are mean values and originate from technical triplicates for each condition, respectively. Culture conditions: 48‐well round well plate, V_L_ = 1000 µL, n = 800 rpm, d_0_ = 3 mm, T = 30°C, X_0_ = 10^6^ spores·ml^–1^, LNP medium with 30 g·L^–1^ glucose

For future investigations and optimization of space‐time yields of secondary metabolite production, it is crucial to understand how and when production is triggered. Here, signaling molecules play a pivotal role. The different γ‐butyrolactones are majorly produced under limiting conditions such as in the early stationary phase. The addition of purified γ‐butyrolactones to *S. coelicolor* was shown to trigger precocious production of actinorhodin and undecylprodigiosin [39]. The presented online monitoring approach would be beneficial for this and could assist in other studies regarding the control mechanisms [40]. Onset time and intensities of the pigment formation could be accurately determined. As a proof of concept, it was tested, whether the addition of supernatant from previous cultivations could manipulate the synchronized pigmentation onset observed in Section 3.2. Therefore, shake flask cultivations were performed and stopped before as well as after pigmentation occurred (Figure S9). The supernatants of these cultivations were sterile filtered and different amounts (1, 5, 10 (v/v) %) were added to new cultivations (Figure 4 and Figure S10). For^1^ reference, 10 (v/v) % deionized water was also added. Since, γ‐butyrolactone concentrations should only be increased in the pigmented supernatants, an earlier pigmentation onset should be observable for these additions. Following the previous findings, the onset of pigmentation is assessed by the declining autofluorescence. The pigmentation for the reference started similarly to preceding results at 115.8 ± 6.0 h (Figure 4 and Figure S10 solid black arrow). The addition of increasing amounts of unpigmented supernatant did not influence the pigmentation onset, accordingly. In contrast to that, the cultures supplemented with pigmented supernatant showed a shortened time until onset of pigmentation, which is notable in the unedited data in Figure S10. The addition of the highest amount of pigmented supernatant with 10 (v/v) % lead to a 27.3 ± 6.6 h precocious pigmentation, compared to the reference. Depending on the type and added amount of supernatant, the autofluorescence increased differently after glucose depletion. This increase was not quantitatively investigated in this study, but our results indicate a link to the phosphate limitation (Figures S1 and S2). Hence, varying amounts of phosphate and metabolites added with the supernatant could explain this pattern. For the precocious pigmentation, Takano et al. discovered a similar result in agar plate tests [39]. Only the addition of transition and stationary phase culture supernatants elicited pigment production in a confluent *S. coelicolor* lawn on agar plates. By testing spent culture supernatant from different media as well as agar types, they also concluded that the nutrient availability had a subordinate role compared to the signaling effect of γ‐butyrolactones. However, in the here presented results, the earlier pigmentation goes along with faster growth. This might be due to the presence of metabolites and building blocks within the added supernatants, which boosted the initial growth of new cultures. The stress signal due to phosphate limitation (Figure 4 and Figure S10) is reached earlier, similarly to the pigmentation onset. Therefore, the difference between these time points remains similar for all conditions. This could be visualized by shifting the data of each condition to align to the stress signal (Figure 4). Aligning the stress signal also resulted in aligning of the pigmentation onset of each condition. Hence, these findings are not an indication for quorum sensing activities, but for a pigmentation onset, which is growth dependent. These results showcase the importance of online monitoring, since these patterns of growth and pigment formation would have been missed by sampling or endpoint determination. The next step would be the addition and testing of purified γ‐butyrolactones to fully elucidate the influence on growth and pigmentation behavior by employing the presented online monitoring system.

## 4 CONCLUDING REMARKS

In this study, small scale online monitoring was presented as a tool to investigate growth and pigment production of complex microbial systems such as *S. coelicolor*. It was demonstrated that the OTR and the autofluorescence signals provide a multitude of information. The autofluorescence, used as a proxy for biomass, also indicates phosphate and glucose depletion as well as pigmentation onset and intensity. It was proven by spectral scattered light measurements that pigment formation led to a clear signal decrease at the respective absorption maximum of the pigments. This allowed precisely timing the onset of pigment formation and estimating pigment quantity non‐invasively either by scattered light or autofluorescence measurements. Under the tested conditions, a rapid change from unpigmented to pigmented cultivations was observed. It could be clearly shown that the rapid change resulted from the pigmentation starting at a fixed time after the phosphate limitation. The pigmentation intensity was mainly dependent on the time frame of carbon availability after pigmentation onset. This was additionally substantiated by investigation of different filling volumes. The filling volume affected the specific power input and therefore, the morphological development. The resulting different carbon consumption rates led to differing times of carbon availability and therefore, differing pigmentation intensities. Quorum sensing was also investigated by the addition of supernatant from previous cultivations. Although, precocious pigment formation was observed up to 27.3 ± 6.6 h earlier, compared to the reference, it could be demonstrated that this was mainly caused by a faster initial growth rather than quorum sensing effects. The cultivation time after phosphate limitation remained the key factor controlling pigmentation onset. The high‐throughput online monitoring and the presented approach enabled the investigation of these effects and interactions, which would otherwise have been missed. As a promising next step, the addition of purified γ‐butyrolactones should be studied with this system.

## CONFLICTS OF INTEREST

The authors have declared no conflicts of interest.

## AUTHORS CONTRIBUTION

Maurice Finger, Fabio Sentek, and Lukas Hartmann planned and carried out the experiments. Maurice Finger processed the experimental data, performed the analysis, drafted the manuscript, and designed the figures. Ana M. Palacio‐Barrera, Ivan Schlembach, Miriam A. Rosenbaum, and Jochen Büchs assisted in data interpretation and participated in drafting the manuscript. Ivan Schlembach, Miriam A. Rosenbaum, and Jochen Büchs supervised the study. All authors read and approved the final manuscript.

## Supporting information



SUPPORTING INFORMATION

## Data Availability

The data that support the findings of this study are available from the corresponding author upon reasonable request.

## Footnotes

1 Layout was updated after publication of the Early View version.
