# Supplementary material for: Insights into Streptomyces coelicolor A3(2) growth and pigment formation with high‐throughput online monitoring
Source: Eng Life Sci. 2022 Apr 28;23(1):e2100151. doi: 10.1002/elsc.202100151 (PMC9815075; doi:10.1002/elsc.202100151)
Supplement: Supplementary file 1 — SUPPORTING INFORMATION [file ELSC-23-e2100151-s001.pdf]

Supplementary material for research article

Insights into *Streptomyces coelicolor* A3(2) growth and pigment formation with high-throughput online monitoring

Maurice Finger[1], Fabio Sentek[1], Lukas Hartmann[1], Ana M. Palacio-Barrera[2,3], Ivan Schlembach[2,3], Miriam A. Rosenbaum[2,3], Jochen Büchs[1]

[1]AVT - Biochemical Engineering, RWTH Aachen University, Aachen, Germany

[2]Leibniz Institute for Natural Product Research and Infection Biology, Hans-Knöll-Institute, Jena, Germany

[3]Faculty of Biological Sciences, Friedrich-Schiller-University, Jena, Germany

Correspondence: Prof. Dr.-Ing. Jochen Büchs (Jochen.Buechs@avt.rwth-aachen.de)

AVT - Biochemical Engineering, RWTH Aachen University, Forckenbeckstraße 51, 52074 Aachen, Germany.

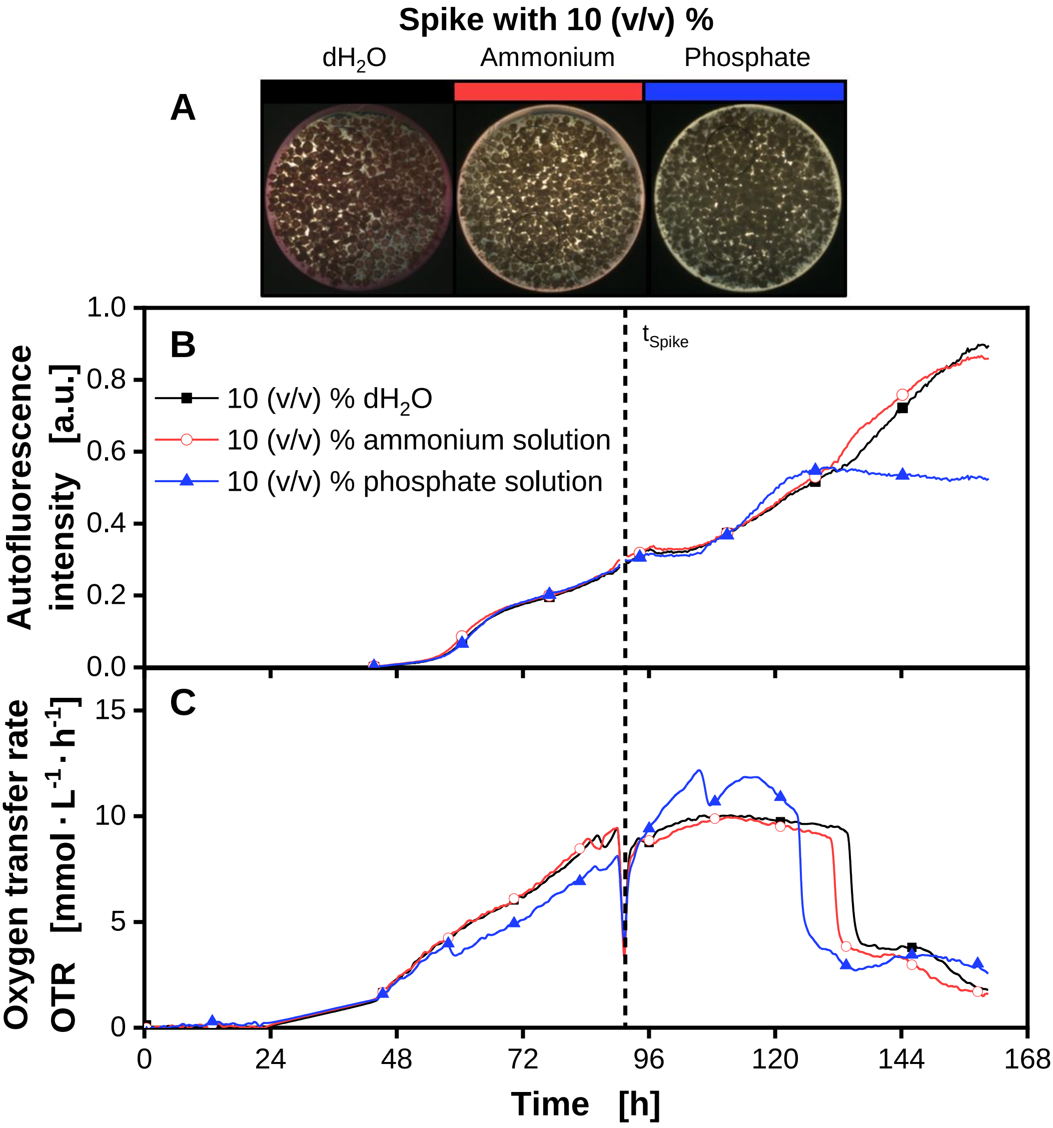

Figure S1. Cultivation of *Streptomyces coelicolor* A3(2) spiked with different solutions at t_{Spike} = 91 h (dashed line). Concentrations for the ammonium and phosphate solution were chosen that the final concentration after spiking resembles the initial medium composition. (**A**) Macroscopic pictures of pellets after termination of the cultivation. The pellet size was in the order of 300 µm. (**B**) Normalized autofluorescence intensity signals (Excitation: 483 nm; Emission: 520 nm). (**C**) Oxygen transfer rates. For clarity, only every 50th data point over time is indicated by the corresponding symbol in Figure S1B and C. Pictures shown in Figure S1A and data presented in Figure S1B and C originate from the same well for each condition, respectively. Culture conditions: 48-well round well plate, V_L = 1000 µL, n = 800 rpm, d_0 = 3 mm, T = 30 °C, X_0 = 10^6 spores·mL^{-1}, LNP medium with 30 g·L^{-1} glucose.

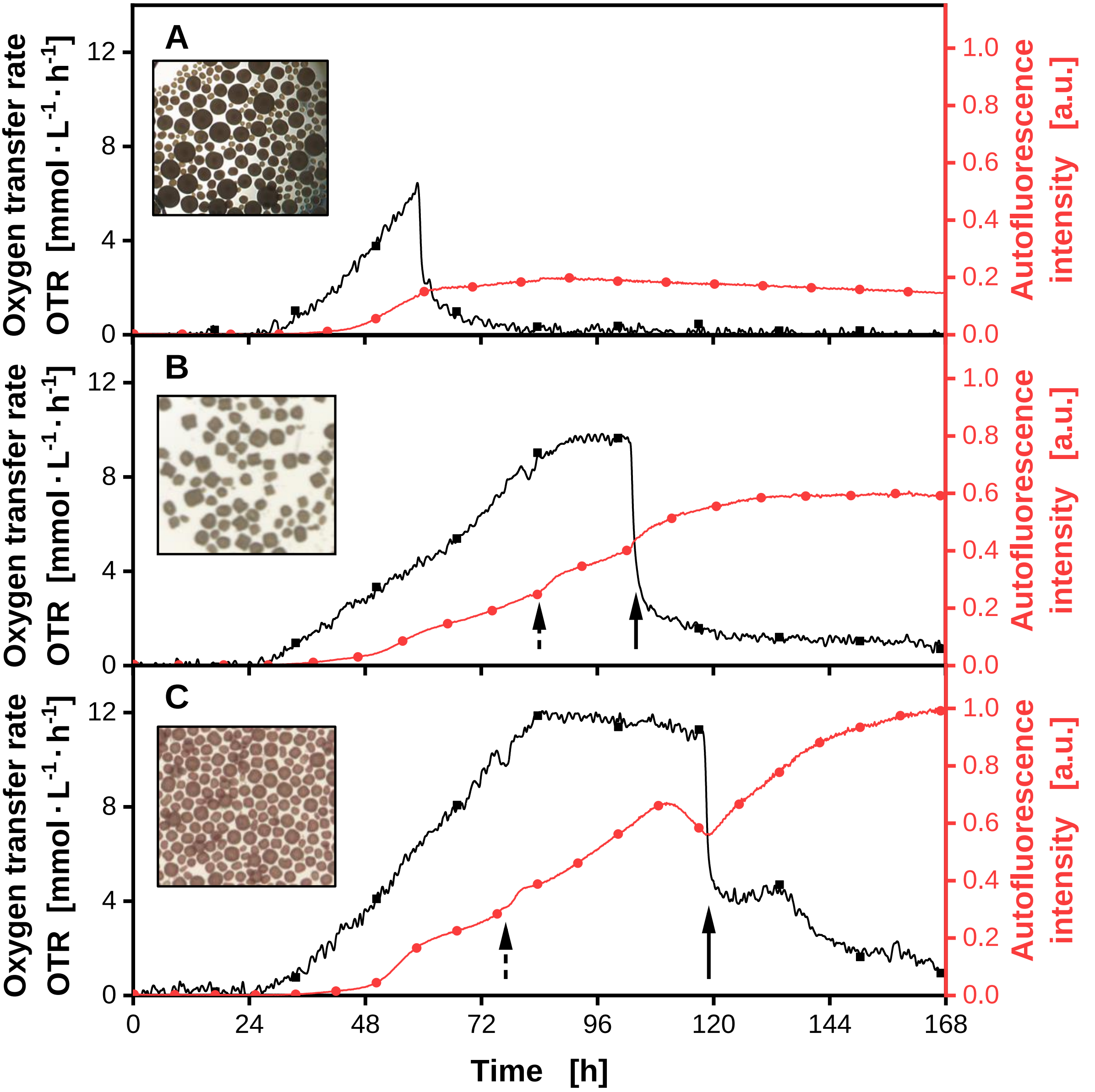

Figure S2. Cultivation of *Streptomyces coelicolor* A3(2) with **(A)** 5 g·L^{-1} glucose (No phosphate limitation and no pigmentation), **(B)** 20 g·L^{-1} glucose (Phosphate limitation and no pigmentation, dataset from Figure 1) and **(C)** 30 g·L^{-1} glucose (Phosphate limitation and pigmentation, dataset from Figure 2). Oxygen transfer rates are depicted in black and normalized autofluorescence intensity signals (Excitation: 483 nm; Emission: 520 nm) in red. Dashed arrows marks phosphate limitation and solid arrows glucose exhaustion. For clarity, only every 50th data point over time is indicated by the corresponding symbol. Culture conditions: 48-well round well plate, V_L = 1000 µL, n = 800 rpm, d_0 = 3 mm, T = 30 °C, X_0 = 10^6 spores·mL^{-1}, LNP medium with varying glucose concentrations.

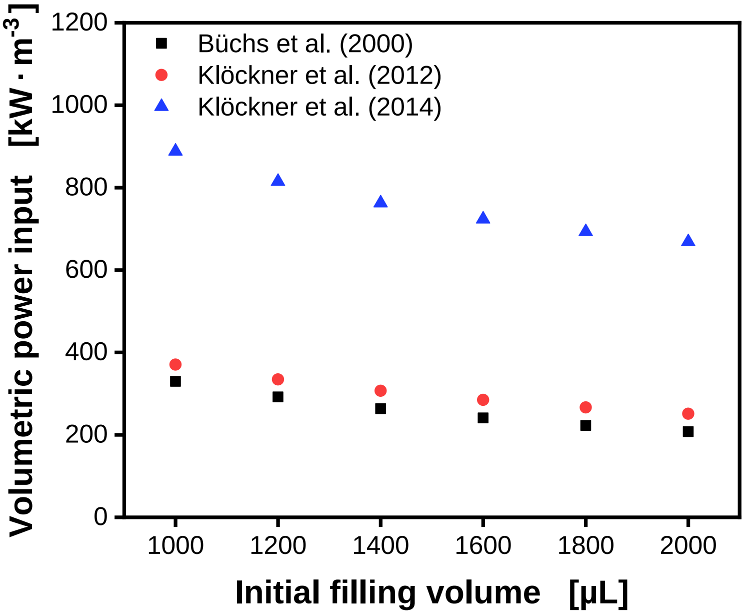

Figure S3. Estimation of volumetric power inputs for varying filling volumes in 48-round well plates with the models of Büchs et al. (2000) for shake flasks, Klöckner et al. (2012) for shaken bioreactors and Klöckner et al. (2014) also for shaken bioreactors [33-35]. Conditions: 48-well round well plate, V_L = 1000 - 2000 µL, n = 800 rpm, d_0 = 3 mm.

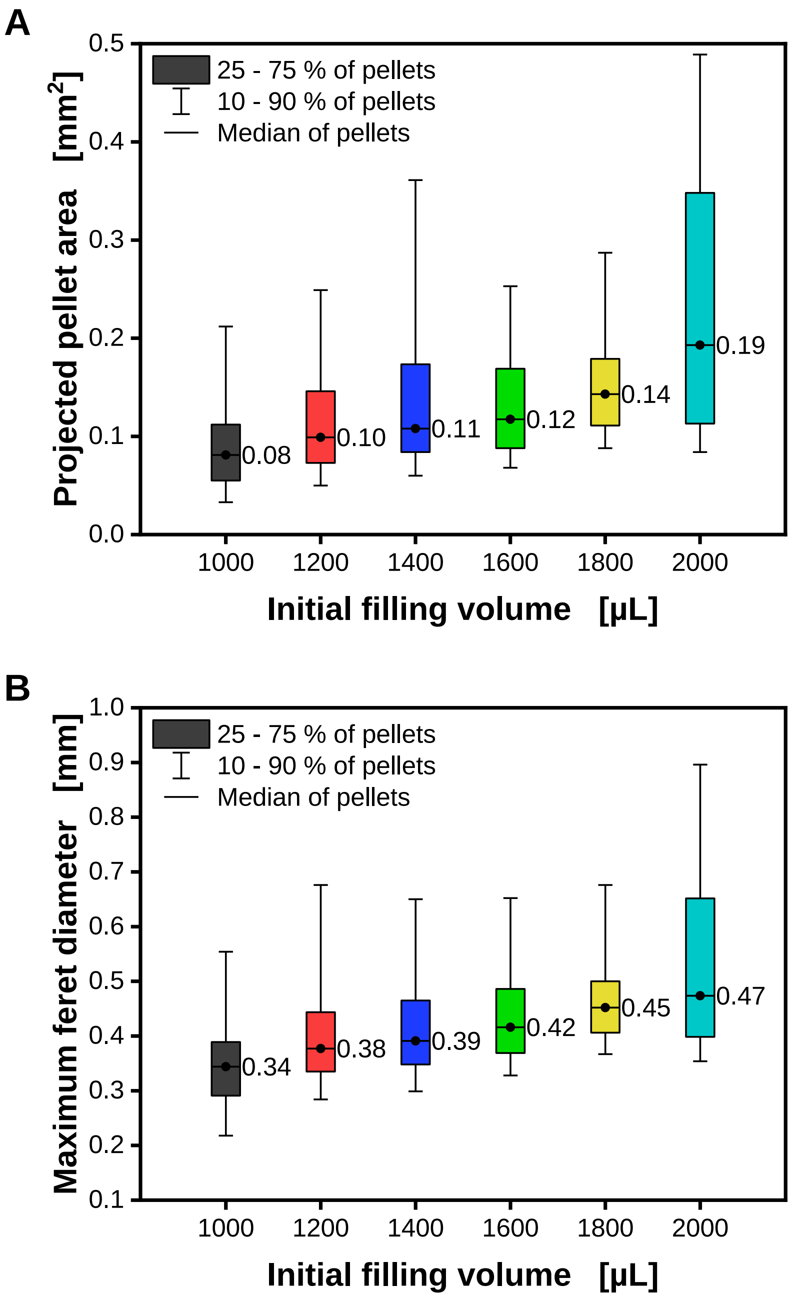

Figure S4. Correlation of the filling volume and the pellet size described by **(A)** projected pellet area and the **(B)** maximum feret diameter. Projected pellet area and maximum ferret diameter were determined by analysis of >50 pellets from the respective pictures (Figure 2A) with the program ImageJ.

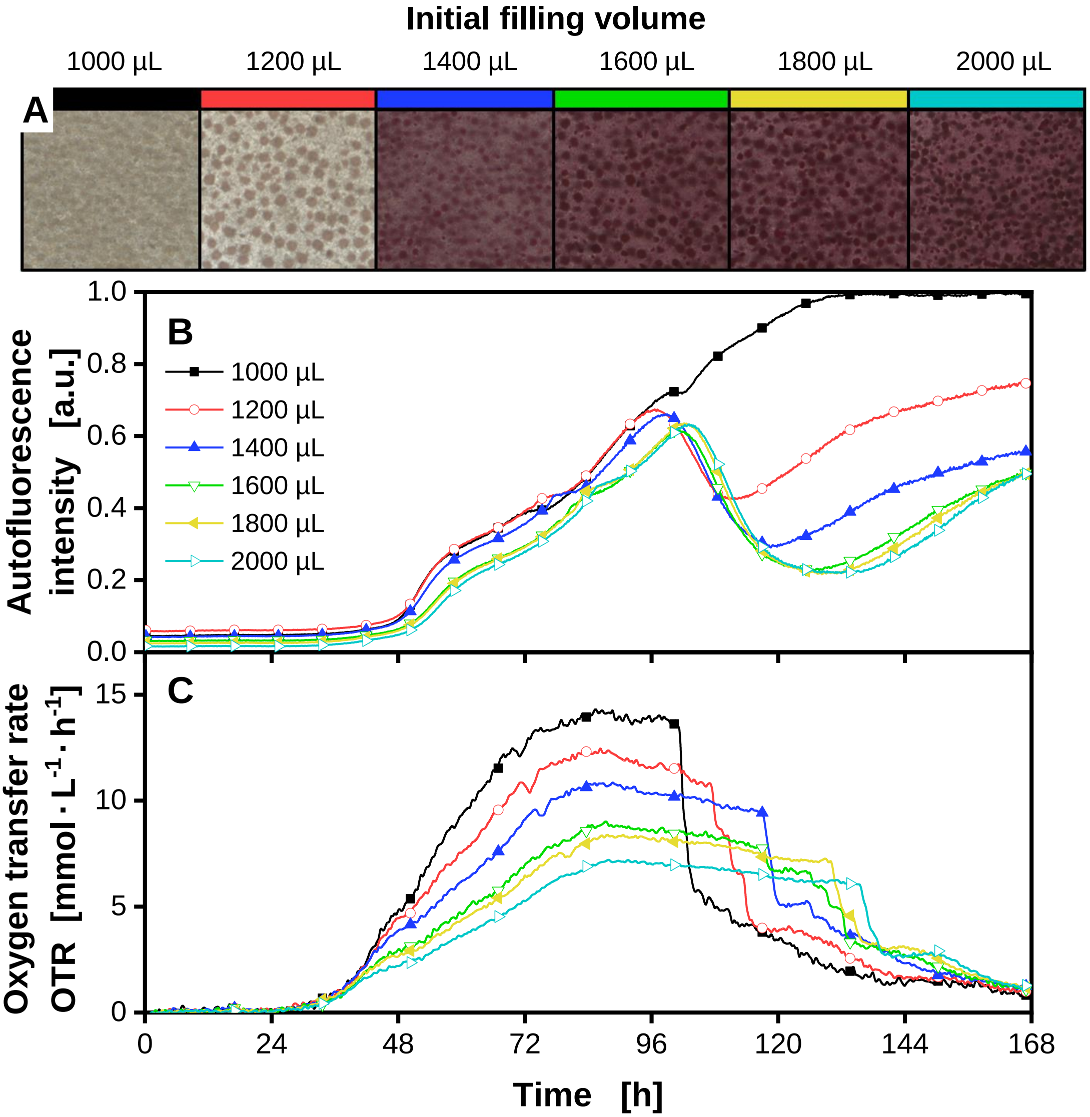

Figure S5. Cultivation of *Streptomyces coelicolor* A3(2) with varying filling volumes, supplemented with 30 mg cellulose per well. (**A**) Macroscopic pictures of pellets after termination of the cultivation. The pellet size was in the order of 300 µm. (**B**) Normalized autofluorescence intensity signals (Excitation: 483 nm; Emission: 520 nm). (**C**) Oxygen transfer rates. For clarity, only every 50th data point over time is indicated by the corresponding symbol in Figure S3B and C. Pictures shown in Figure S3A and data presented in Figure S3B and C originate from the same well for each condition, respectively. Culture conditions: 48-well round well plate, 30 mg cellulose per well, V_L = 1000 - 2000 µL, n = 800 rpm, d_0 = 3 mm, T = 30 °C, X_0 = 10^6 spores·mL^{-1}, LNP medium with 30 g·L^{-1} glucose.

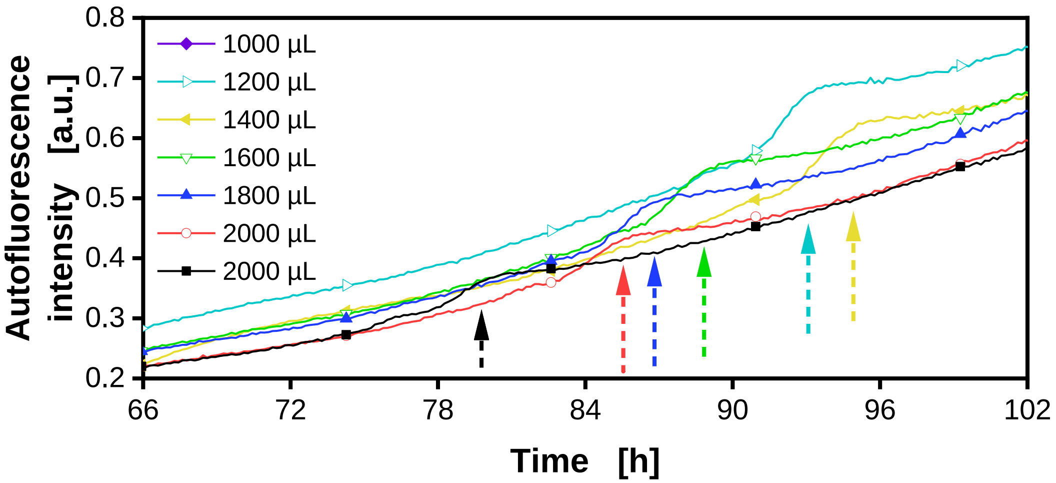

Figure S6. Enlarged version of figure 2 from 66 – 102 h. Cultivation of *Streptomyces coelicolor* A3(2) with varying filling volumes. Normalized autofluorescence intensity signals (Excitation: 483 nm; Emission: 520 nm). Dashed arrows mark the stress signal due to phosphate limitation. For clarity, only every 50th data point over time is indicated by the corresponding symbol. Culture conditions: 48-well round well plate, V_L = 1000 - 2000 µL, n = 800 rpm, d_0 = 3 mm, T = 30 °C, X_0 = 10^6 spores·mL^{-1}, LNP medium with 30 g·L^{-1} glucose.

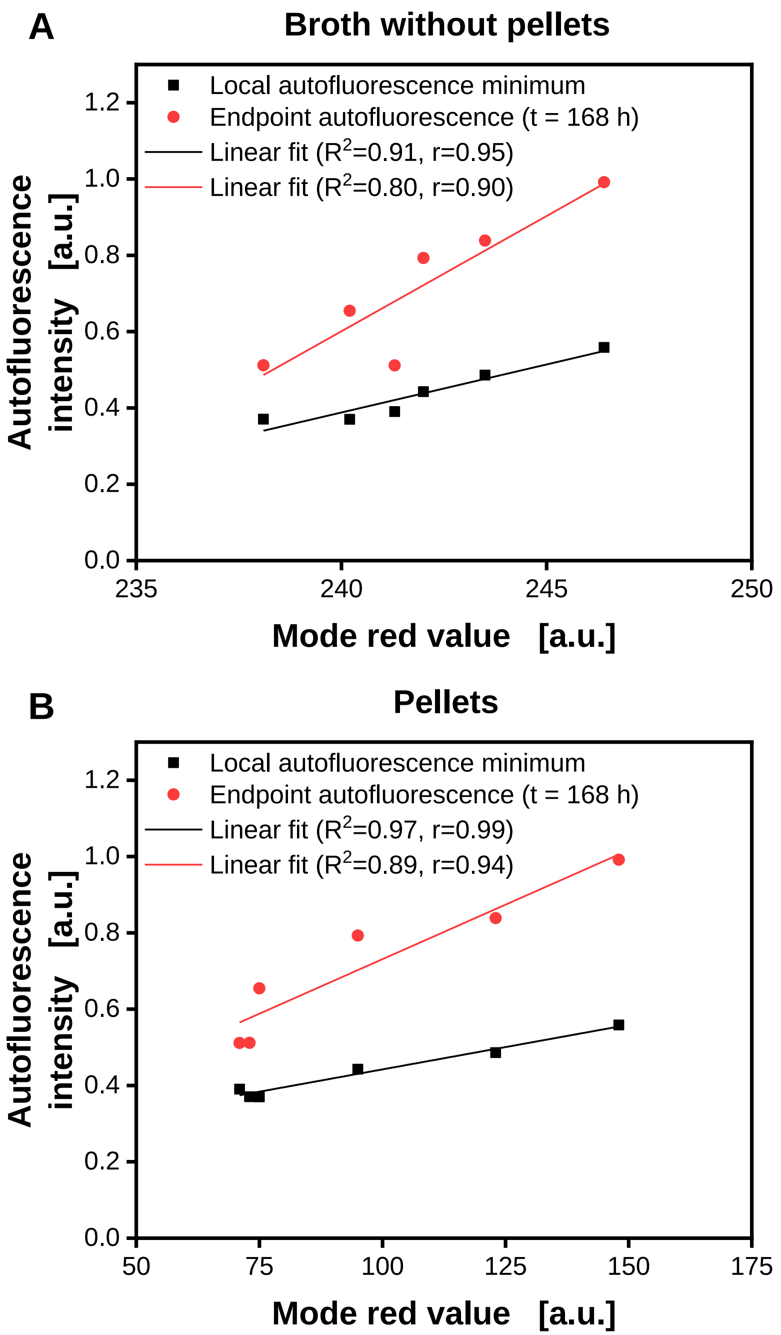

Figure S7. Correlation of the autofluorescence signals (Excitation: 483 nm; Emission: 520 nm) from the dataset of Figure 2 with the pigmentation intensity represented by the mode red value. The autofluorescence intensities were taken at the local minimum when glucose exhaustion occurred and from the endpoint at t = 168 h. The mode red value for the **(A)** culture broth (background) and the **(B)** pellets was determined by analysis of >5000 pixels from the respective pictures in Figure 2A with the program ImageJ.

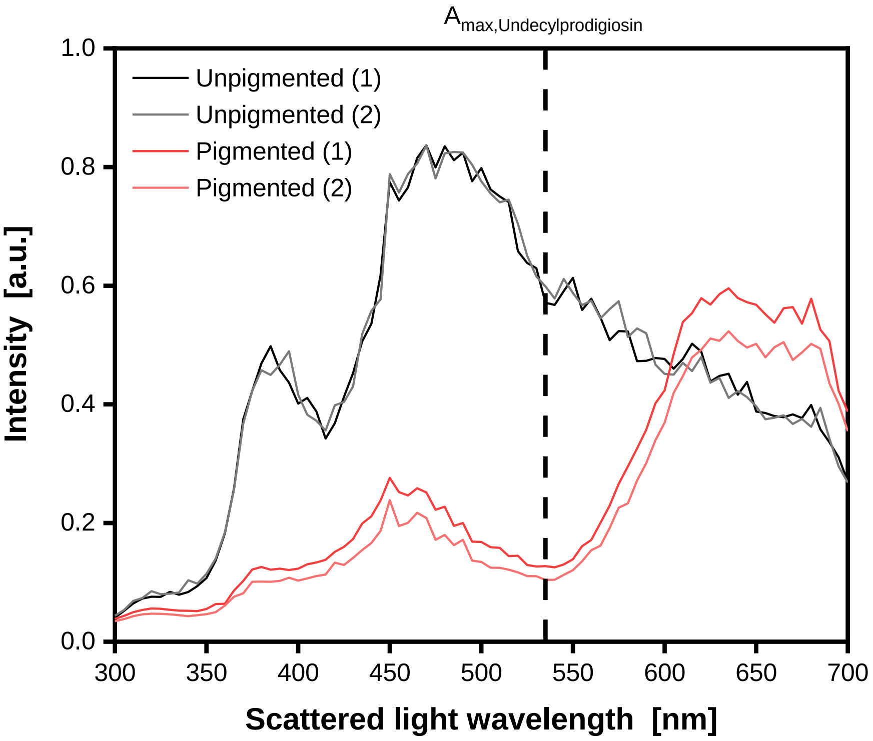

Figure S8. Comparison of scattered light signals of pigmented and unpigmented *Streptomyces coelicolor* A3(2) samples in duplicates taken from shake flask cultivations similarly to the samples from S5. Maximum absorption wavelength of undecylprodigiosin is indicated by the dashed line [37] Conditions: 48-well round well plate, V_L = 1000 µL, n = 800 rpm, d_0 = 3 mm, T = 30 °C.

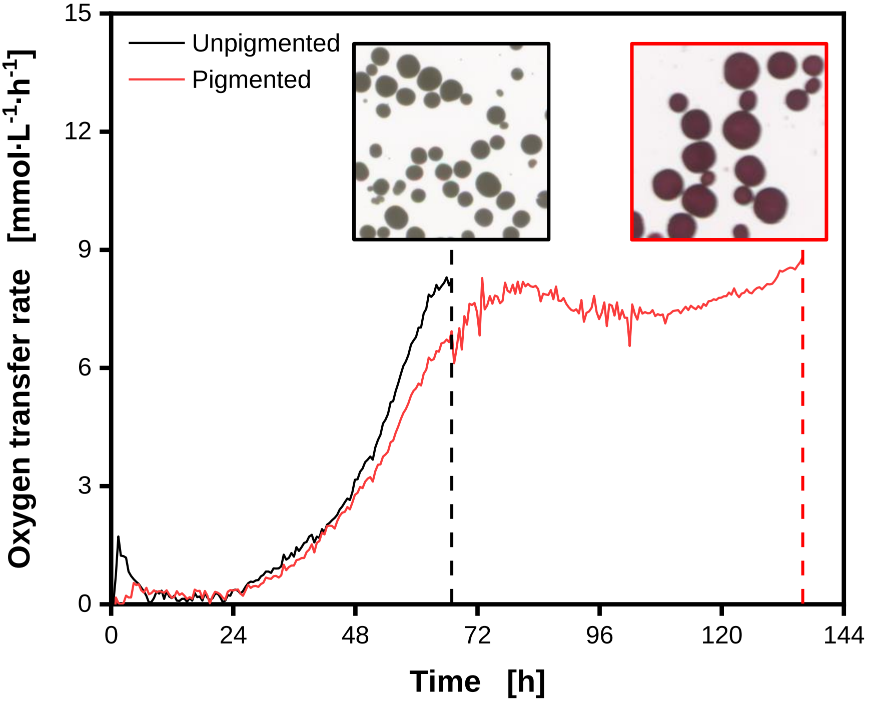

Figure S9. Cultivation of *Streptomyces coelicolor* A3(2) in shake flasks. Termination of cultivations are indicated by dashed lines. From these macroscopic pictures for unpigmented and pigmented conditions were taken and supernatant for supplementing was generated. Culture conditions: 250 mL shake flasks, V_L = 20 mL, n = 350 rpm, d_0 = 50 mm, T = 30 °C, X_0 = 10^6 spores·mL^{-1}, LNP medium with 30 g·L^{-1} glucose.

A

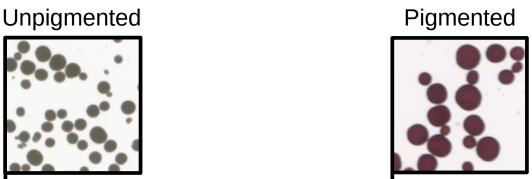

	Reference	1 (v/v) % unpigmented	5 (v/v) % unpigmented	10 (v/v) % unpigmented	1 (v/v) % pigmented	5 (v/v) % pigmented	10 (v/v) % pigmented
$t_{Limitation}$ [h]	91.3 ± 1.8	84.8 ± 7.0	84.8 ± 0.1	85.4 ± 0.6	72.3 ± 2.3	65.9 ± 0.6	66.7 ± 0.5
$t_{Pigmentation}$ [h]	115.8 ± 6.0	109.1 ± 8.4	107.1 ± 2.4	112.5 ± 0.6	95.9 ± 3.1	91.0 ± 1.1	88.5 ± 0.6
**Difference** [h]	24.5 ± 7.8	24.3 ± 15.4	23.3 ± 2.5	27.1 ± 1.2	23.6 ± 5.4	25.1 ± 1.7	21.8 ± 1.1

B

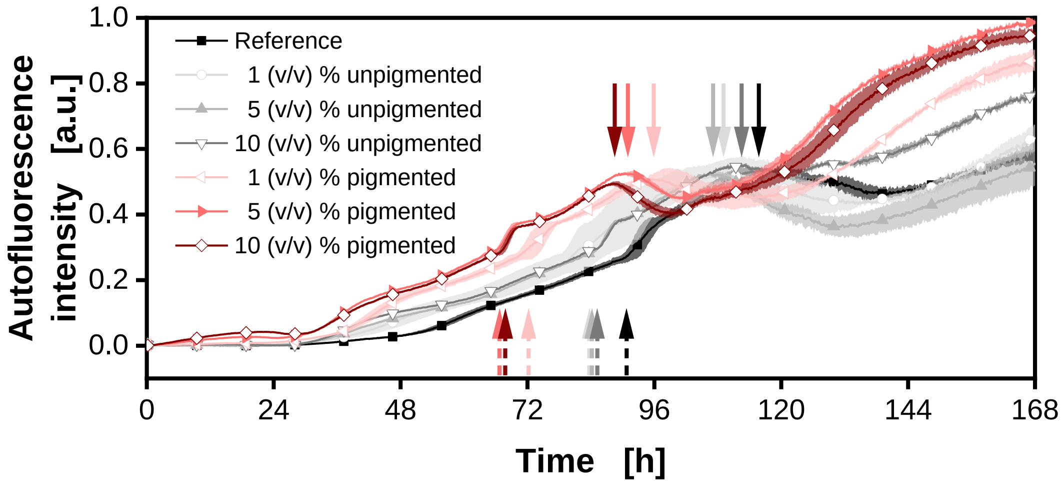

Figure S10. Cultivation of *Streptomyces coelicolor* A3(2) supplemented with different amounts of supernatants from unpigmented (grey scale) and pigmented (red scale) cultures. (**A**) Macroscopic pictures of the shake flasks cultivations from which the supernatant was taken. The added volumetric amount is stated in (v/v) %. The resulting time points of the stress signal due to phosphate limitation $t_{Limitation}$ and pigmentation onset $t_{Pigmentation}$ as well as the difference is given. (**B**) Normalized autofluorescence intensity signals (Excitation: 483 nm; Emission: 520 nm). Dashed arrows mark $t_{Limitation}$ and solid arrows mark $t_{Pigmentation}$. For clarity, only every 50th data point over time is indicated by the corresponding symbol. Data presented in Figure S10A and B are mean values and originate from technical triplicates for each condition, respectively. Shaded areas represent standard deviations. Culture conditions: 48-well round well plate, V_L = 1000 µL, n = 800 rpm, d_0 = 3 mm, T = 30 °C, $X_0 = 10^6$ spores·mL^{-1}, LNP medium with 30 g·L^{-1} glucose.
